# Demographic science aids in understanding the spread and fatality rates of COVID-19

**DOI:** 10.1073/pnas.2004911117

**Published:** 2020-04-16

**Authors:** Jennifer Beam Dowd, Liliana Andriano, David M. Brazel, Valentina Rotondi, Per Block, Xuejie Ding, Yan Liu, Melinda C. Mills

**Affiliations:** ^a^Leverhulme Centre for Demographic Science, Nuffield College, University of Oxford, Oxford OX1 3UQ, United Kingdom

**Keywords:** COVID-19, demography, age structure, mortality

## Abstract

Governments around the world must rapidly mobilize and make difficult policy decisions to mitigate the coronavirus disease 2019 (COVID-19) pandemic. Because deaths have been concentrated at older ages, we highlight the important role of demography, particularly, how the age structure of a population may help explain differences in fatality rates across countries and how transmission unfolds. We examine the role of age structure in deaths thus far in Italy and South Korea and illustrate how the pandemic could unfold in populations with similar population sizes but different age structures, showing a dramatically higher burden of mortality in countries with older versus younger populations. This powerful interaction of demography and current age-specific mortality for COVID-19 suggests that social distancing and other policies to slow transmission should consider the age composition of local and national contexts as well as intergenerational interactions. We also call for countries to provide case and fatality data disaggregated by age and sex to improve real-time targeted forecasting of hospitalization and critical care needs.

Governments are rapidly mobilizing to minimize transmission of coronavirus disease 2019 (COVID-19) through social distancing and travel restrictions to reduce fatalities and outstripping of healthcare capacity. The pandemic’s progression and impact are strongly related to the demographic composition of the population, specifically, population age structure. Demographic science can provide new insights into how the pandemic may unfold and the intensity and type of measures needed to slow it down. Currently, COVID-19 mortality risk is highly concentrated at older ages, particularly those aged 80+ y. In China, case fatality rate (CFR) estimates range from 0.4% for those 40 y to 49 y jumping to 14.8% for those 80+ y (1). This age pattern has been even more stark in Italy, where, as of March 30, 2020, the reported CFR is 0.7% for those 40 y to 49 y, and 27.7% for those >80 y, with 96.9% of deaths occurring in those aged 60 y and over (2). Current CFRs are likely overestimated due to underascertainment of cases. In South Korea, with broader testing and strong health care capacity (only 158 deaths), the current CFR for those 80+ y is still an alarming 18.31% (3).

## The Importance of Age Structure for COVID-19 Transmission and Fatality Rates

Population age structure may explain the remarkable variation in fatalities across countries and the vulnerability of Italy. The deluge of fatal COVID-19 cases in Italy was unexpected, given the affected region’s health and wealth. Italy is one of the oldest populations, with 23.3% of its population over 65 y, compared to 12% in China (4). Italy is also characterized by extensive intergenerational contacts, supported by a high degree of residential proximity between adult children and parents (5). Even when intergenerational families do not coreside, daily contacts are frequent. Many Italians prefer to live close to extended family, with over half of the population in the northern regions commuting (6). Intergenerational interactions, coresidence, and commuting may have accelerated the outbreak in Italy through social networks that increased the proximity of elderly to initial cases (7).

The age structure of initial cases, along with early detection and treatment, likely explains the low numbers of fatalities in South Korea and Germany. The Korean outbreak was concentrated among the young Shincheonji religious group (3), with only 4.5% of cases thus far falling into the >80-y group (8). This contributed to a low overall CFR in South Korea relative to Italy (1.6% vs. 10.6%). Germany has, likewise, few deaths (583 out of 61,923 cases to date), with the median age of confirmed cases at 48 y compared to 62 y in Italy (9). COVID-19 transmission chains that begin in younger populations may go undetected longer (10), with countries slow to raise the alarm. The initial low CFR in England may have reflected the relatively young age structure of early infections, including Greater London, which has a small fraction of residents over 65 y compared to more rural areas (11). COVID-19 was only detected in King County, WA, once it reached the Life Care Center in Kirkland, where 19 out of 22 of the state's first reported COVID-19 deaths occurred, with virus genetic sequence estimates suggesting it circulated for several weeks prior (12). Once community transmission is established, countries with high intergenerational contacts may see faster transmissions to high-fatality age groups, as seen in Italy and Spain, leading to higher average CFR (13). The overall burden of serious cases and mortality reflects linkages between the age distribution of early cases, age structure of the population, and intergenerational connections.

**Fig. 1.**
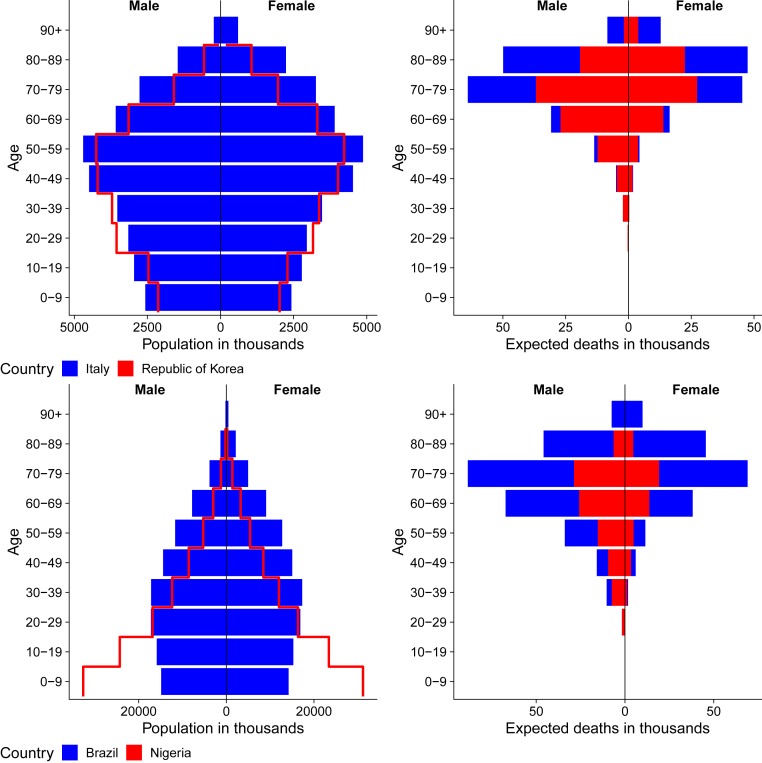
Population composition (*Left*) and expected deaths in population (*Right*) for Italy and Republic of Korea (*Top*) and Nigeria and Brazil (*Bottom*). Projections assume 10% population infection rate and current age−sex-specific case fatality rates from Italy (Dataset S1).

**Fig. 2.**
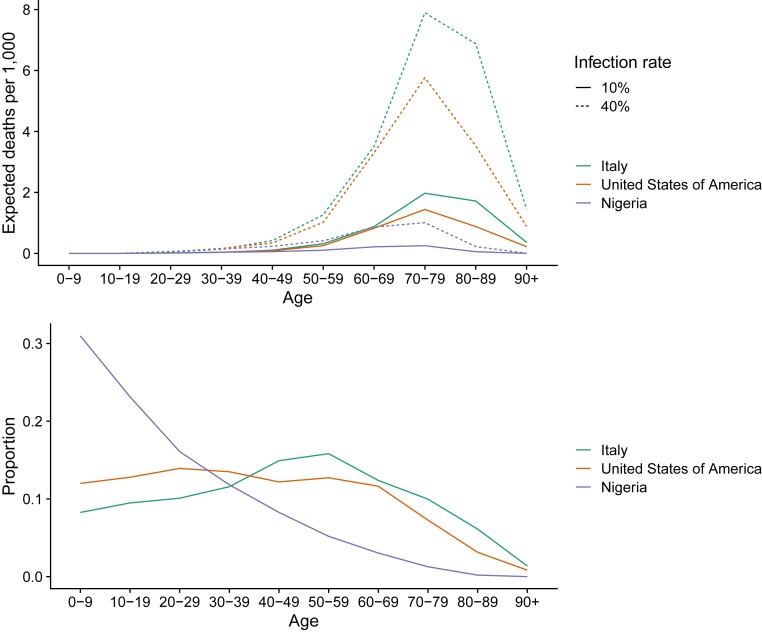
Expected deaths by total population (*Top*) and proportion of total population by age group (*Bottom*) for Italy, United States, and Nigeria, with different levels of population infection and current age-specific fatality rates from Italy.

Fig. 1 contains population pyramids to illustrate how population age structure interacts with high COVID-19 mortality rates at older ages to generate large differences across populations in the number of deaths, holding constant assumed rates of infection prevalence (10%) and age−sex-specific CFRs (Italy) (14). Adjusting assumptions changes the total number of expected deaths but not the relative comparisons across countries with different age structures. For example, assuming that CFRs, by age, are half of current Italian rates would reduce the numbers of expected deaths by half. Fig. 1, *Top* considers two countries, Italy and South Korea, with very different population age structures. The larger number of expected fatalities is clearly visible in Fig. 1, *Top Right* for Italy (302,530) versus Korea (177,822). In Fig. 1, *Bottom*, we consider two countries with similar population sizes but very different age distributions. Brazil has 2.0% of its population over age 80+ y, with our simulated scenario leading to dramatically more deaths (452,694) compared to Nigeria (142,056), where only 0.2% are 80+ y. Fig. 2 visualizes expected deaths by age groups in countries with different population age structures: Italy (older), United States (middle), and Nigeria (younger). We see stark implications of an older age structure for higher fatalities, amplified at higher population infection rates. *SI Appendix*, Fig. S1 animates differences by infection rate (0 to 100%).

## Demographic Science and COVID-19 Policy

Demographically informed projections will better predict the COVID-19 burden and inform governments. While population age structure is crucial for understanding those at the highest risk of mortality both across and within countries, it is also vital for understanding social distancing measures to reduce critical cases that overload the health system—aka “flattening the curve.” Our illustrations show that countries with older populations must take aggressive protective measures. For these to be effective, special attention should be devoted to high-risk population groups and intergenerational contact. Within countries, mapping of age-related spatial clustering can improve hospital and critical care forecasts (15).

Consideration of population age structure also necessitates understanding the interlinkage of policy measures and how policies might create unintended consequences. While schools may be a hub of virus transmission, school closures may inadvertently bring grandparents and children into contact if grandparents become the default carers. In aged populations with close intergenerational ties, governments need to facilitate childcare solutions that reduce contact. In a pending decree, the Italian government introduced a special leave for parents with children at home from school, and a voucher for babysitting.

The age structure of populations also suggests that the squeezed “sandwich” generation of adults who care for both the old and young are important for mitigating transmission. Beyond introducing sick pay for those who need to self-isolate or care for family members, joint government and industry emergency policy measures should seek to counter family economic crises, particularly for vulnerable and precarious workers who are less able to comply with policies that allow social distancing.

The rapid spread of COVID-19 has revealed the need to understand how population dynamics interact with pandemics. Population aging is currently more pronounced in wealthier countries, which, mercifully, may lessen the impact of this pandemic in lower-income countries with weaker health systems but younger age structures. It is plausible that poor general health status and coinfections such as HIV and tuberculosis will increase the danger of COVID-19 in these countries, along with intergenerational proximity and challenges to physical distancing. Thus far, the lower than expected number of cases detected in Africa (despite extensive trade and travel links with China) suggests that the young age structure may be protective of severe and thus detectable cases. Beyond age structure, demography can shed light on the large sex differences in COVID-19 mortality that need to be understood—with men at higher risk. Distributions of underlying comorbidities such as diabetes, hypertension, and chronic obstructive pulmonary disease will likewise refine risk estimates. Until more nuanced data are available, the concentration of mortality risk in the oldest old ages remains one of the best tools to predict the burden of critical cases and produce more precise planning of availability of hospital beds, staff, and other resources. Few countries are routinely releasing their COVID-19 data with key demographic information such age, sex, or comorbidities. We call for the timely release of these disaggregated data to allow researchers and governments to nowcast risk for more focused prevention and preparedness.

## Methods

### Data and Analysis.

Data to produce Figs. 1 and 2 are from https://population.un.org/wpp (4), and age−sex-specific case fatality rates are from Italian data (https://www.epicentro.iss.it/coronavirus/bollettino/Bollettino-sorveglianza-integrata-COVID-19_30-marzo-2020.pdf), accessed March 30, 2020 (2). For Figs. 1 and 2, total number of expected deaths by age group was derived by multiplying the total number of people in each age−sex group and country by an assumed population infection rate of 0.1 (and 0.4 for Fig. 2) and Italian age−sex-specific fatality rates (Movie S1) (March 30, 2020). Data analysis is in R using the packages (ggplot2).

## Supplementary Material

Supplementary File

Supplementary File

Supplementary File
