# Supplementary material for: Demographic science aids in understanding the spread and fatality rates of COVID-19
Source: Proc Natl Acad Sci U S A. 2020 Apr 16;117(18):9696–8. doi: 10.1073/pnas.2004911117 (PMC7211934; doi:10.1073/pnas.2004911117)
Supplement: Supplementary File [file pnas.2004911117.sapp.pdf]

PNAS
www.pnas.org

Supplementary Information for

Demographic science aids in understanding the spread and fatality rates of COVID-19

Jennifer Beam Dowd*, Liliana Andriano, Valentina Rotondi, David M. Brazel, Per Block, Xuejie Ding, Yan Liu, Melinda C. Mills*
Leverhulme Centre for Demographic Science, University of Oxford & Nuffield College, UK

*Corresponding authors email: jennifer.dowd@sociology.ox.ac.uk and melinda.mills@nuffield.ox.ac.uk

Other supplementary materials for this manuscript include the following:

Movie S1

www.pnas.org/cgi/doi/10.1073/pnas.2004911117

Movie S1 (separate file). Animation of Expected deaths by total population (per 1,000) and proportion of total population by age group, Italy, United States of America, Nigeria, with varying levels of infection rates from 0% to 100%.
