# Supplementary figures and images for: Demographic science aids in understanding the spread and fatality rates of COVID-19
Source: Proc Natl Acad Sci U S A. 2020 Apr 16;117(18):9696–8. doi: 10.1073/pnas.2004911117 (PMC7211934; doi:10.1073/pnas.2004911117)

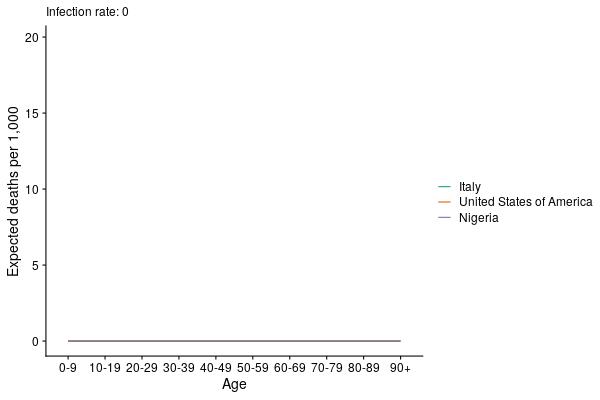

Supplement: Supplementary File [file pnas.2004911117.sm01.gif]
